# Supplementary material for: Ground-dwelling invertebrate diversity in domestic gardens along a rural-urban gradient: Landscape characteristics are more important than garden characteristics
Source: PLoS One. 2020 Oct 2;15(10):e0240061. doi: 10.1371/journal.pone.0240061 (PMC7531831; doi:10.1371/journal.pone.0240061)
Supplement: S1 Table — P-values are given in brackets. (DOCX) [file pone.0240061.s001.docx]

**S1 Table. Species richness correlations^1^ among taxonomical groups within 35 gardens (Pearson correlations (R) or Spearman correlations (R_s_)).** P-values are given in brackets.

|  | Gastropods | Spiders | Spiders suppl. | Millipedes | Woodlice^2^ | Ants | Ground beetles^2^ | Rove beetles^2^ | Beetles combined | Total species richness | Total species richness suppl. |
| --- | --- | --- | --- | --- | --- | --- | --- | --- | --- | --- | --- |
| Gastropods |  | R = 0.24  (0.16) | R = 0.16  (0.35) | R = -0.12  (0.49) | R_s_ = 0.18  (0.30) | R = 0.18  (0.30) | R_s_ = 0.22  (0.32) | R_s_ = 0.10  (0.56) | R = 0.22  (0.21) | **R = 0.70**  **(< 0.0001)** | **R = 0.70**  **(< 0.0001)** |
| Spiders | R = 0.24  (0.16) |  | **R = 0.94**  **(< 0.0001)** | R = 0.15  (0.40) | R_s_ = -0.16  (0.36) | **R = 0.35**  **(0.0372)** | R_s_ = -0.17  (0.13) | R_s_ = 0.12  (0.48) | R = 0.00  (0.98) | **R = 0.43**  **(0.0097)** | **R = 0.44**  **(0.0079)** |
| Spiders suppl. | R = 0.16  (0.35) | **R = 0.94**  **(< 0.0001)** |  | R = 0.11  (0.54) | R_s_ = -0.23  (0.19) | **R = 0.36**  **(0.0343)** | R_s_ = -0.26  (0.10) | R_s_ = -0.02  (0.89) | R = -0.13  (0.46) | R = 0.28  (0.0992) | R = 0.33  (0.0522) |
| Millipedes | R = -0.12  (0.49) | R = 0.15  (0.40) | R = 0.11  (0.54) |  | R_s_ = 0.06  (0.74) | R = -0.04  (0.80) | R_s_ = -0.05  (0.76) | R_s_ = -0.06  (0.74) | R = -0.15  (0.38) | R = 0.11  (0.46) | R = 0.10  (0.58) |
| Woodlice^2^ | R_s_ = 0.18  (0.30) | R_s_ = -0.16  (0.36) | R_s_ = -0.16  (0.36) | R_s_ = 0.06  (0.74) |  | R_s_ = -0.12  (0.51) | R_s_ = 0.22  (0.20) | R_s_ = 0.12  (0.51) | R_s_ = 0.21  (0.22) | **R_s_ = 0.35**  **(0.0413)** | R_s_ = 0.31  (0.0672) |
| Ants | R = 0.18  (0.30) | **R = 0.35**  **(0.0372)** | **R = 0.36**  **(0.0343)** | R = -0.04  (0.80) | R_s_ = -0.12  (0.51) |  | R_s_ = -0.21  (0.22) | R_s_ = 0.01  (0.88) | R = -0.01  (0.95) | **R = 0.46**  **(0.0056)** | **R = 0.48**  **(0.0033)** |
| Ground beetles^2^ | R_s_ = 0.22  (0.32) | R_s_ = -0.17  (0.13) | R_s_ = -0.26  (0.10) | R_s_ = -0.05  (0.76) | R_s_ = 0.22  (0.20) | R_s_ = -0.21  (0.22) |  | R_s_ = 0.15  (0.39) | **R_s_ = 0.41**  **(0.0137)** | R_s_ = 0.30  (0.0797) | R_s_ = 0.28  (0.10) |
| Rove beetles^2^ | R_s_ = 0.10  (0.56) | R_s_ = 0.12  (0.48) | R_s_ = -0.02  (0.89) | R_s_ = -0.06  (0.74) | R_s_ = 0.12  (0.51) | R_s_ = 0.01  (0.88) | R_s_ = 0.15  (0.39) |  | **R_s_ = 0.94**  **(< 0.0001)** | **R_s_ = 0.55**  **(0.0006)** | **R_s_ = 0.52**  **(0.0013)** |
| Beetles combined | R = 0.22  (0.21) | R = 0.00  (0.98) | R = -0.13  (0.46) | R = -0.15  (0.38) | R_s_ = 0.21  (0.22) | R = -0.01  (0.95) | **R_s_ = 0.41**  **(0.0137)** | **R_s_ = 0.94**  **(< 0.0001)** |  | **R = 0.67**  **(< 0.0001)** | **R = 0.65**  **(< 0.0001)** |
| Total species richness | **R = 0.70**  **(< 0.0001)** | **R = 0.43**  **(0.0097)** | R = 0.28  (0.0992) | R = 0.11  (0.46) | **R_s_ = 0.35**  **(0.0413)** | **R = 0.46**  **(0.0056)** | R_s_ = 0.30  (0.0797) | **R_s_ = 0.55**  **(0.0006)** | **R = 0.67**  **(< 0.0001)** |  | **R = 0.99**  **(< 0.0001)** |
| Total species richness suppl. | **R = 0.70**  **(< 0.0001)** | **R = 0.44**  **(0.0079)** | R = 0.33  (0.0522) | R = 0.10  (0.58) | R_s_ = 0.31  (0.0672) | **R = 0.48**  **(0.0033)** | R_s_ = 0.28  (0.10) | **R_s_ = 0.52**  **(0.0013)** | **R = 0.65**  **(< 0.0001)** | **R = 0.99**  **(< 0.0001)** |  |

1 Data on species richness was log-transformed before analysis

2 Spearman correlations based on untransformed data were used for analyses involving woodlice, ground beetles and rove beetles as species richness of these taxonomical groups was not normally distributed even after transformation.

Significant values are indicated in bold.
