# Supplementary material for: Ground-dwelling invertebrate diversity in domestic gardens along a rural-urban gradient: Landscape characteristics are more important than garden characteristics
Source: PLoS One. 2020 Oct 2;15(10):e0240061. doi: 10.1371/journal.pone.0240061 (PMC7531831; doi:10.1371/journal.pone.0240061)
Supplement: S2 Table — (DOCX) [file pone.0240061.s002.docx]

**S2 Table. Means and ranges for garden and landscape characteristics (n = 35 in all cases).**

|  | **Unit** | **Mean (SE)** | **Median (range)** |
| --- | --- | --- | --- |
| **Garden size** | | | |
| Total garden area | m^2^ | 479.5 (53.1) | 432 (61–1379) |
| Area with vegetation | m^2^ | 412.1 (48.2) | 367.1 (28.8–1276.9) |
| Grassland area | m^2^ | 165.6 (27.0) | 132.4 (4.0–752.3) |
| Percentage of grassland | % | 37.1 (3.1) | 35.6 (3.7–66.8) |
| Area of shrubs and trees | m^2^ | 74.9 (13.2) | 68.6 (0.1–276.7) |
| Percentage shrub and tree cover | % | 16.9 (2.6) | 13.5 (0.1–57.9) |
| **Garden habitat diversity** | | | |
| Habitat richness | count | 7.7 (0.2) | 8 (4–9) |
| Structural diversity | Shannon index | 3.7 (0.1) | 3.8 (2.6–4.4) |
| **Naturalness** | | | |
| Total native plant species richness | count | 57.2 (4.7) | 52 (14–128) |
| Native plant species richness in grassland | count | 32.1 (2.7) | 31 (8–80) |
| **Isolation of gardens** | | | |
| Length of non-permeable garden border | m | 36.2 (4.9) | 30.0 (0.0–104.7) |
| Percentage length of non-permeable garden border | % | 37.3 (4.5) | 31.9 (0.0–95.6) |
| Index of permeable garden border | % | 59.8 (4.4) | 62.3 (4.4–100.0) |
| **Landscape characteristics** | | | |
| Percentage of sealed area | % | 52.9 (2.3) | 52.6 (32.8–87.0) |
| Percentage of green area | % | 45.4 (2.5) | 46.8 (6.8–67.2) |
| Distance to city centre | m | 3307.4 (369.1) | 2864 (556–9516) |
