# Supplementary figures and images for: Ground-dwelling invertebrate diversity in domestic gardens along a rural-urban gradient: Landscape characteristics are more important than garden characteristics
Source: PLoS One. 2020 Oct 2;15(10):e0240061. doi: 10.1371/journal.pone.0240061 (PMC7531831; doi:10.1371/journal.pone.0240061)

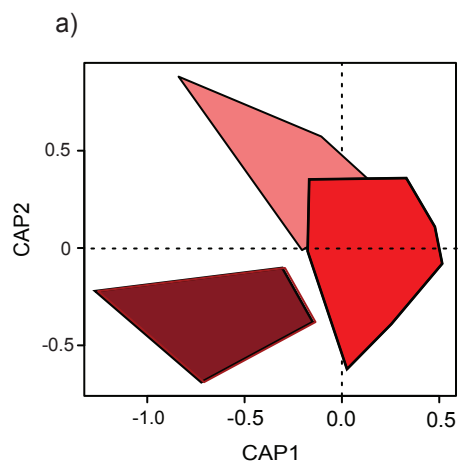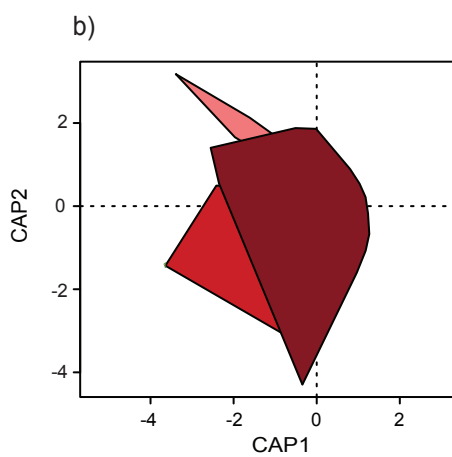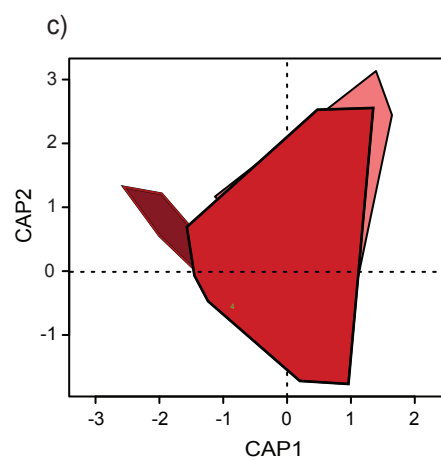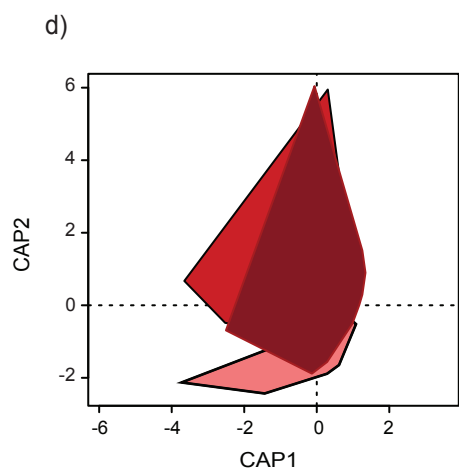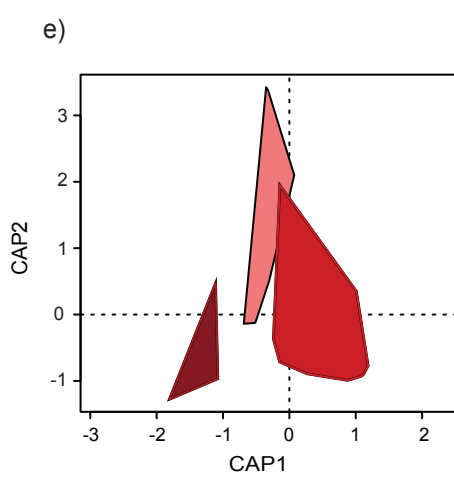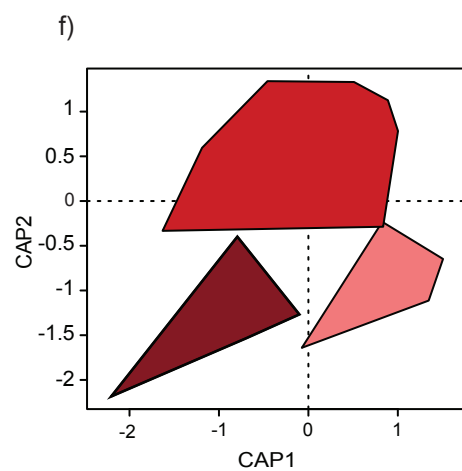

Supplement: S1 Fig — Results of constrained analyses of principle coordinates visualizing similarities in species compositions of gastropods (a), spiders (b), millipedes (c), woodlice (d), ants (e), and rove beetles (f) in gardens with different percentages of sealed area within a radius of 200 m (three classes). Dark red refers to gardens with a high percentage of sealed area, red to gardens with intermediate percentage of sealed area and light red to gardens with low percentage of sealed area in the surroundings. (PDF) [file pone.0240061.s008.pdf]

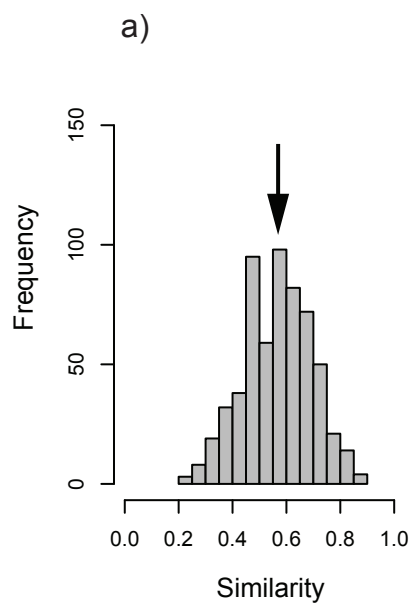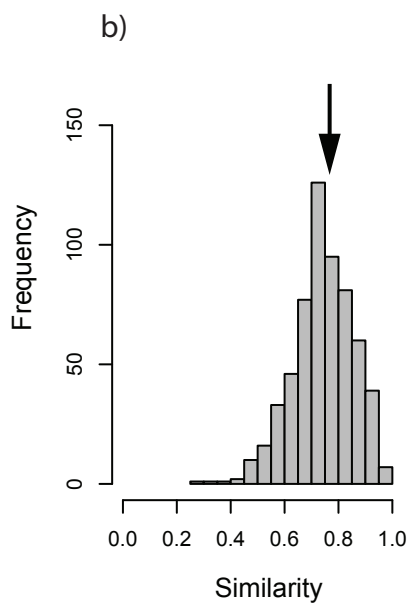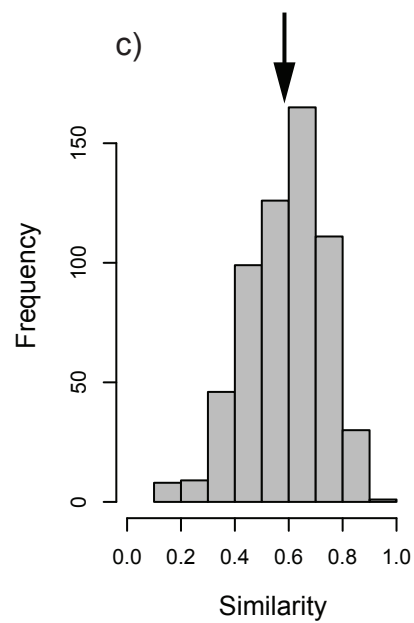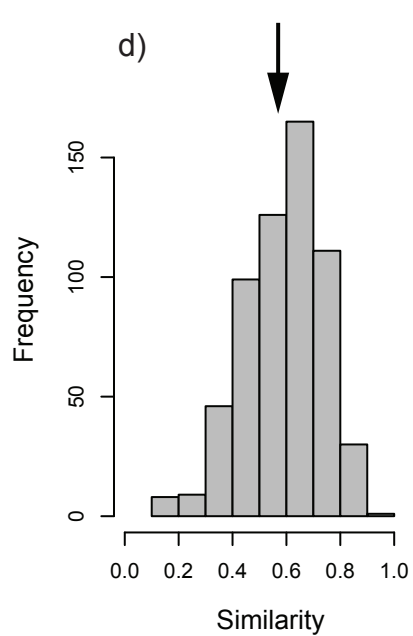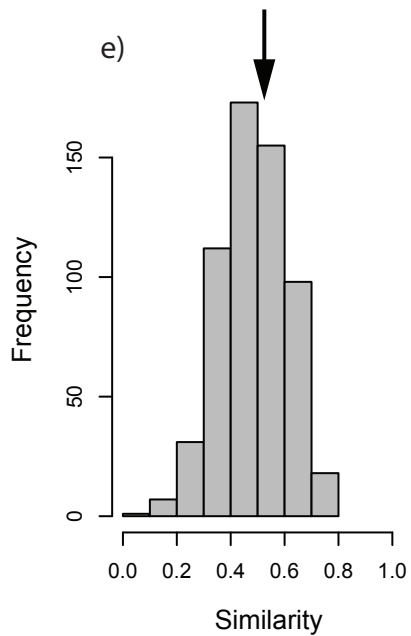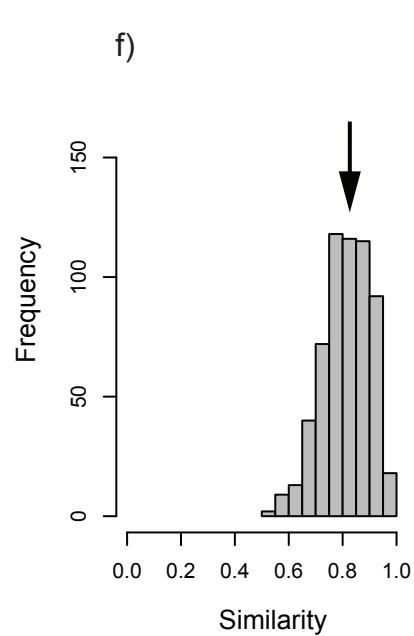

Supplement: S2 Fig — Distribution of Sørensen-indices of species compositions obtained from all combinations of each two gardens for gastropods (a), spiders (b), millipedes (c), woodlice (d), ants (e), and rove beetles (f). (PDF) [file pone.0240061.s009.pdf]

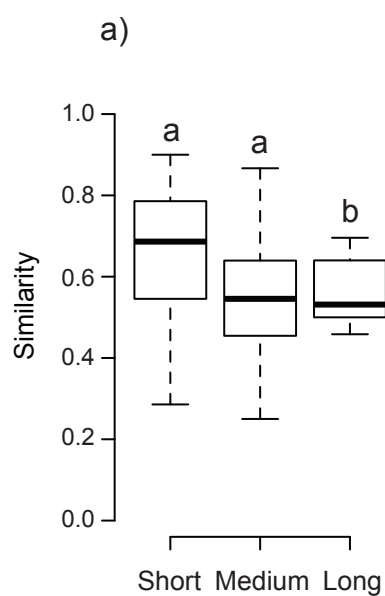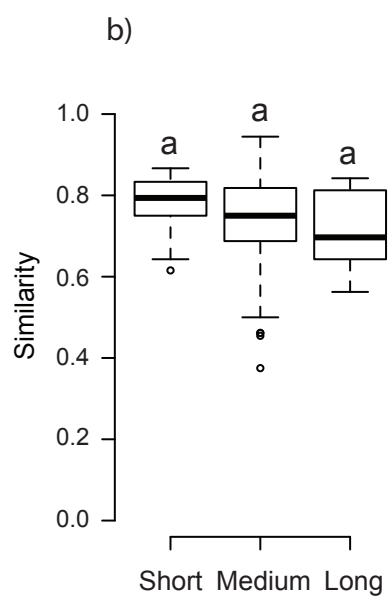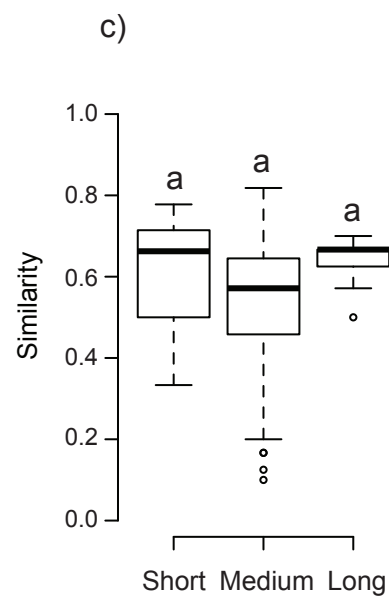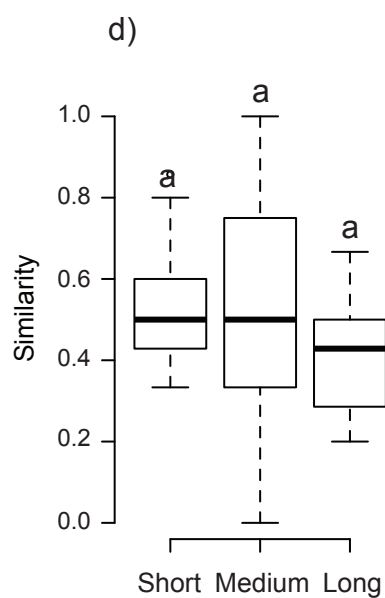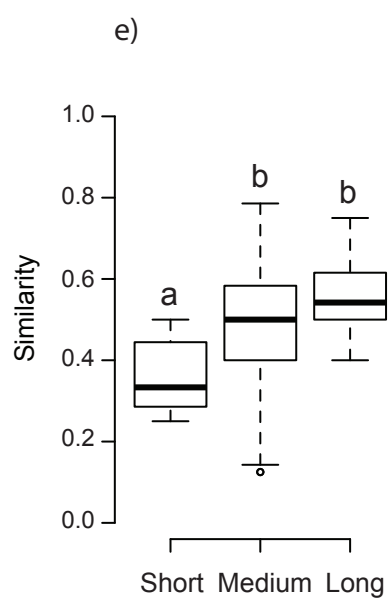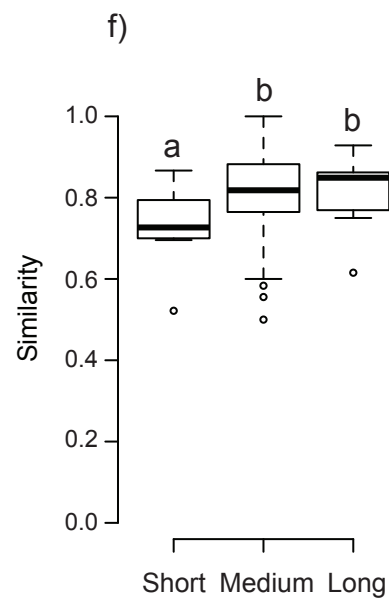

Supplement: S3 Fig — Effects of distance to the city centre (three classes) on the Sørensen-indices of species compositions of gastropods (a), spiders (b), millipedes (c), woodlice (d), ants (e), and rove beetles (f). The Sørensen-indices of species compositions were calculated for all combinations of each two gardens belonging to the same distance class. Different letters indicate significant differences among distance classes (Tukey’s HSD, P < 0.05). (PDF) [file pone.0240061.s010.pdf]

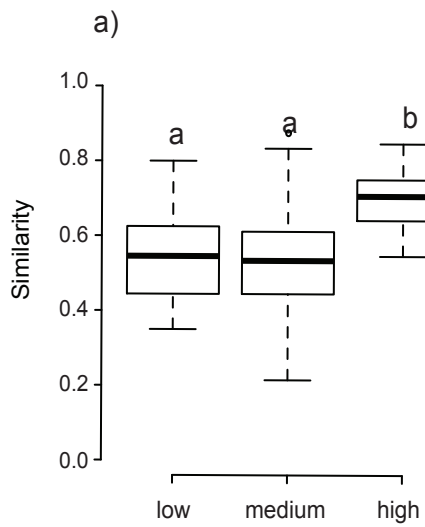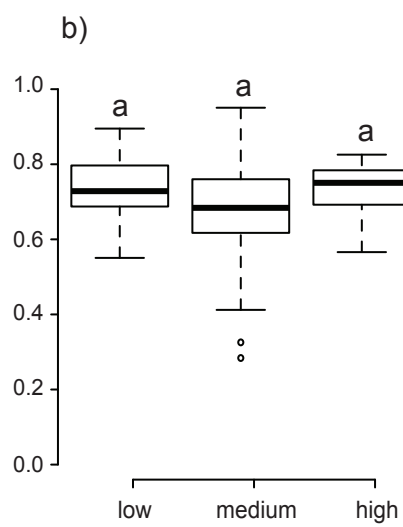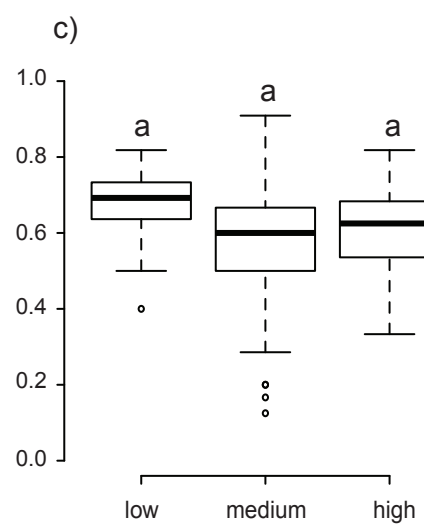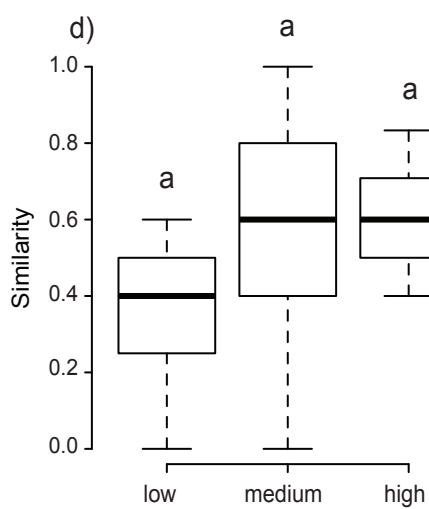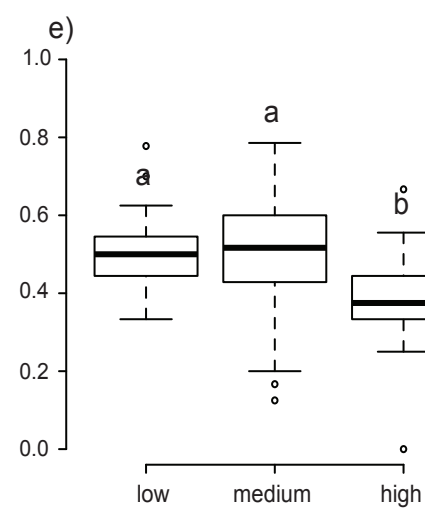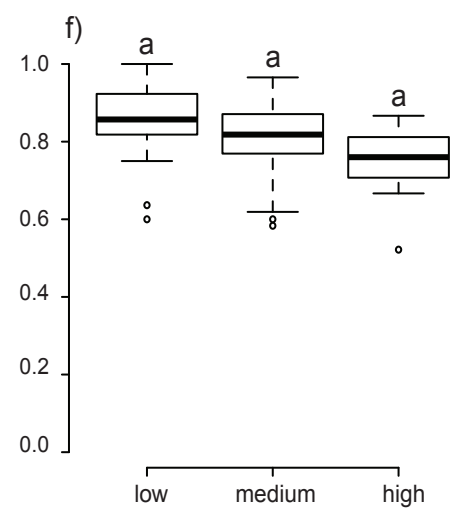

Supplement: S4 Fig — Effects of percentage of sealed area within a radius of 200 m (three classes) on the Sørensen-indices of species compositions of gastropods (a), spiders (b), millipedes (c), woodlice (d), ants (e), and rove beetles (f). The Sørensen-indices of species compositions were calculated for all combinations of each two gardens belonging to the same distance class. Different letters indicate significant differences among distance classes (Tukey’s HSD, P < 0.05). (PDF) [file pone.0240061.s011.pdf]
